# Evaluation of an ultraviolet LED trap for catching *Anopheles* and *Culex* mosquitoes in south-eastern Tanzania

**DOI:** 10.1186/s13071-019-3673-7

**Published:** 2019-08-27

**Authors:** Emmanuel P. Mwanga, Halfan S. Ngowo, Salum A. Mapua, Arnold S. Mmbando, Emmanuel W. Kaindoa, Khamis Kifungo, Fredros O. Okumu

**Affiliations:** 1 Environmental Health and Ecological Sciences Department, Ifakara Health Institute, Morogoro, Tanzania; 2 Institute of Biodiversity, Animal Health and Comparative Medicine, University of Glasgow, Glasgow, UK; 3 School of Public Health, University of Witwatersrand, Johannesburg, South Africa

**Keywords:** Malaria, *An. arabiensis*, *An. funestus*, *Culex* spp., Mosclean trap, Ifakara Health Institute, Light-emitting diodes (LEDs)

## Abstract

**Background:**

Improved surveillance techniques are required to accelerate efforts against major arthropod-borne diseases such as malaria, dengue, filariasis, Zika and yellow-fever. Light-emitting diodes (LEDs) are increasingly used in mosquito traps because they improve energy efficiency and battery longevity relative to incandescent bulbs. This study evaluated the efficacy of a new ultraviolet LED trap (Mosclean) against standard mosquito collection methods.

**Methods:**

The study was conducted in controlled semi-field settings and in field conditions in rural south-eastern Tanzania. The Mosclean trap was compared to commonly used techniques, namely CDC-light traps, human landing catches (HLCs), BG-Sentinel traps and Suna traps.

**Results:**

When simultaneously placed inside the same semi-field chamber, the Mosclean trap caught twice as many *Anopheles arabiensis* as the CDC-light trap, and equal numbers to HLCs. Similar results were obtained when traps were tested individually in the chambers. Under field settings, Mosclean traps caught equal numbers of *An. arabiensis* and twice as many *Culex* mosquitoes as CDC-light traps. It was also better at trapping malaria vectors compared to both Suna and BG-Sentinel traps, and was more efficient in collecting mosquitoes indoors than outdoors. The majority of *An. arabiensis* females caught by Mosclean traps were parous (63.6%) and inseminated (89.8%). In comparison, the females caught by CDC-light traps were 43.9% parous and 92.8% inseminated.

**Conclusions:**

The UV LED trap (Mosclean trap) was efficacious for sampling *Anopheles* and *Culex* mosquitoes. Its efficacy was comparable to and in some instances better than traps commonly used for vector surveillance. The Mosclean trap was more productive in sampling mosquitoes indoors compared to outdoors. The trap can be used indoors near human-occupied nets, or outdoors, in which case additional CO_2_ improves catches. We conclude that this trap may have potential for mosquito surveillance. However, we recommend additional field tests to validate these findings in multiple settings and to assess the potential of LEDs to attract non-target organisms, especially outdoors.

## Background

The World Health Organization, through its Global Technical Strategy for Malaria Elimination (GTS) [1] and Global Vector Control Response initiative (GVCR) [2], has called for strengthening and integration of surveillance as a core component of strategies against mosquito-borne diseases. To operationalize this agenda, endemic countries need low-cost and scalable monitoring tools, as well as a simplified set of indicators for surveillance. For malaria and other vector-bone diseases, surveillance plays a major role in: (i) tracking transmission; (ii) assessing susceptibility of vectors to interventions; (iii) measuring receptivity in specific locations; and (iv) predicting disease outbreaks [3, 4]. Various traps have been developed and used for mosquito sampling and surveillance, often for experimental studies but also for programmatic purposes [5–7]. Some of the traps have also been considered as control intervention when used as mosquito trapping [8–10]. A major setback for many of the existing techniques is poor scalability due to their physical structure and cost.

The human landing catch (HLC) is the most direct and scalable method for measuring human biting rates [11] and is regarded as the gold standard for collecting host-seeking mosquitoes [7, 12–15]. However, using humans directly as bait to collect mosquitoes has multiple limitations. For example, this technique: (i) is expensive in large-scale operations; (ii) exposes humans to mosquito bites and thus increases the risk of infections in field settings; (iii) is labor-intensive; and (iv) needs close supervision, high skills and motivation [16]. To address these shortcomings, new ways of safely carrying out HLCs have been proposed and tested, e.g. the human-baited double net (HDN) and mosquito electrocuting grid trap (MET) [17, 18]. Another common trap is the Center for Disease Prevention and Control light trap (CDC-light trap), improved by Sudia & Chamberlain [19] and widely used for indoor collections of host-seeking *Anopheles* [20, 21]. The CDC-light trap uses incandescent light bulbs, battery cells and a motor-driven fan, all of which make it expensive and difficult to maintain in many settings. Despite these challenges, the CDC-light trap is still considered one of the simplest trapping techniques, requiring only light as an attractant.

Recent developments in trapping technologies and improved understanding of mosquito olfactory systems have resulted in several new trapping devices for malaria vectors. Some of these exploit the olfactory behaviors and biting preferences of mosquitoes, e.g. BG-Sentinel trap, Suna trap, Ifakara tent trap and BG-malaria [22–24]. The BG-Sentinel trap and Suna trap are simple and portable tools which can be used to sample day-biting and night-biting mosquitoes including *Anopheles* and *Aedes* [23, 25, 26]. Others, such as the Ifakara tent trap, allow exposure-free sampling in rural and urban settings [14, 24]. One drawback of these traps is that they usually require synthetic lures, such as Ifakara lure [27], Mbita lures (MB5) [28] or carbon dioxide gas (CO_2_) to mimic human odors. Others such as the Ifakara tent trap and human-baited double net traps, are large, bulky and inconveniently require human volunteers as bait.

Light-emitting diodes (LEDs) are also increasingly being tested for arthropod trapping. Compared to standard incandescent light bulbs used in CDC-light traps, the LED technology provides: (i) higher energy efficiency; (ii) longer battery life; (iii) lower costs per unit time of trapping; (iv) options for specific wavelengths targeting different arthropod species; and (v) greater trapping efficiencies.

CDC-light traps have previously been modified by replacing the incandescent bulbs with LEDs emitting blue, green or red light to achieve higher trapping efficiencies [29–33]. For example, Costa-Neta et al. [29] showed that LED lamps emitting blue (470 nm) or green (520 nm) light consistently caught high numbers of *Anopheles* mosquitoes, regardless of the lunar cycles. Silva et al. [31] also showed the superiority of green-LEDs over incandescent light for catching different species of sand flies in Brazil. Elsewhere, in Egypt, red-LEDs attracted more *Phlebotomus papatasi* sand flies than either blue or green LEDs or incandescent bulbs [32]. Lastly, tests on woodland mosquitoes in Florida revealed differential attractiveness of species to LEDs of different colors [33].

The Mosclean trap is a simple LED trap which utilizes ultraviolet (UV) emitting diodes combined with a titanium dioxide (TiO_2_) plate for photocatalytic conversion to produce CO_2_. The trap is created by Sensor Electronic Technology Inc. (Columbia, SC, USA) and Seoul Viosys (Gyeonggi-do, Republic of Korea), using proprietary UV LED technology (violeds™). It has been previously demonstrated as highly efficacious for trapping *Aedes albopictus* and *Aedes aegypti* mosquitoes in the USA [34], but to our knowledge has yet to be tested against *Anopheles* mosquitoes anywhere.

The aim of the present study was to evaluate the efficacy of the Mosclean trap in catching malaria vectors and other mosquitoes in south-eastern Tanzania in comparison to the CDC-light trap, BG-Sentinel trap and Suna trap.

## Methods

### Description of the semi-field system and study areas for field experiments

Experiments were conducted under both semi-field and field settings. All semi-field tests were done inside a large multi-chambered screen house (28.8 × 21 m) at the Ifakara Health Institute [35], Ifakara, Tanzania. We used a compartment measuring 9.6 × 21 m [35] in which there were two experimental huts with a peri-domestic area consisting of different vegetation types and pebbles, to mimic local housing and ecological setup [36].

**Fig. 1:**
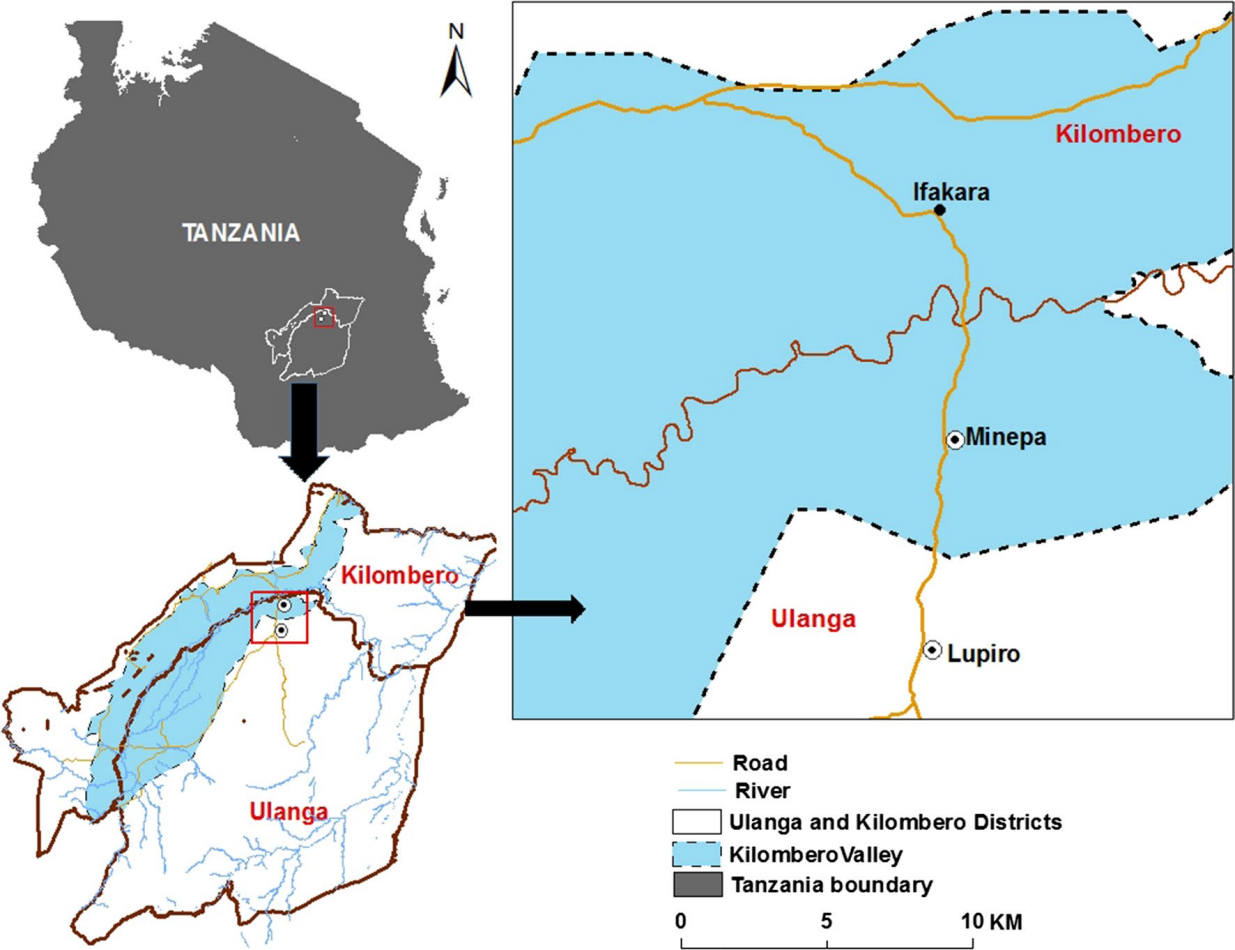
Map showing study villages in Ulanga district, south-eastern Tanzania (courtesy of Alex J. Limwagu)

The field tests were conducted in villages in Ulanga District, south-eastern Tanzania, approximately 20 km south of Ifakara town (Fig. 1). The area lies on the Kilombero flood plains between the Udzungwa Mountains in the north and the Mahenge hills to the south. The main economic activities include agriculture (mostly rice and maize cultivation), fishing and small-scale trade. Annual rainfall ranges from 1200 to 1800 mm and temperatures from 22 to 32.6 °C. Short rains occur from November to December, while long rains occur between March and June.

### Mosquitoes

The mosquitoes used were from colonies of the Ifakara strain of *Anopheles arabiensis*, originally established in 2009 with specimens from Lupiro village, approximately 25 km south of Ifakara town. The colony is maintained at 27 ± 2 °C and 75 ± 10% relative humidity (RH) as previously described by Batista et al. [37]. Larvae are reared in plastic basins and fed twice a day on Tetramin® fish food (Tetra GmbH, Melle, Germany). Adults are fed on 10% glucose solution. Colony maintenance also involved *ad libitum* blood meals given to females *via* human volunteer arms. The field tests, however, targeted free-flying wild mosquitoes of different species from the study villages.

### Mosquito traps and lures

The main candidate test trap was the Mosclean trap, which emits 365 nm UV using violeds™ technology and generates CO_2_ gas *via* a photocatalytic reaction on the TiO_2_ plate surfaces. The trap measures 20 cm (diameter) and 28.8 cm (height) and runs on either DC or AC current. They can operate with small portable solar cells to charge a portable power pack for use in places with no electricity.

**Fig. 2:**
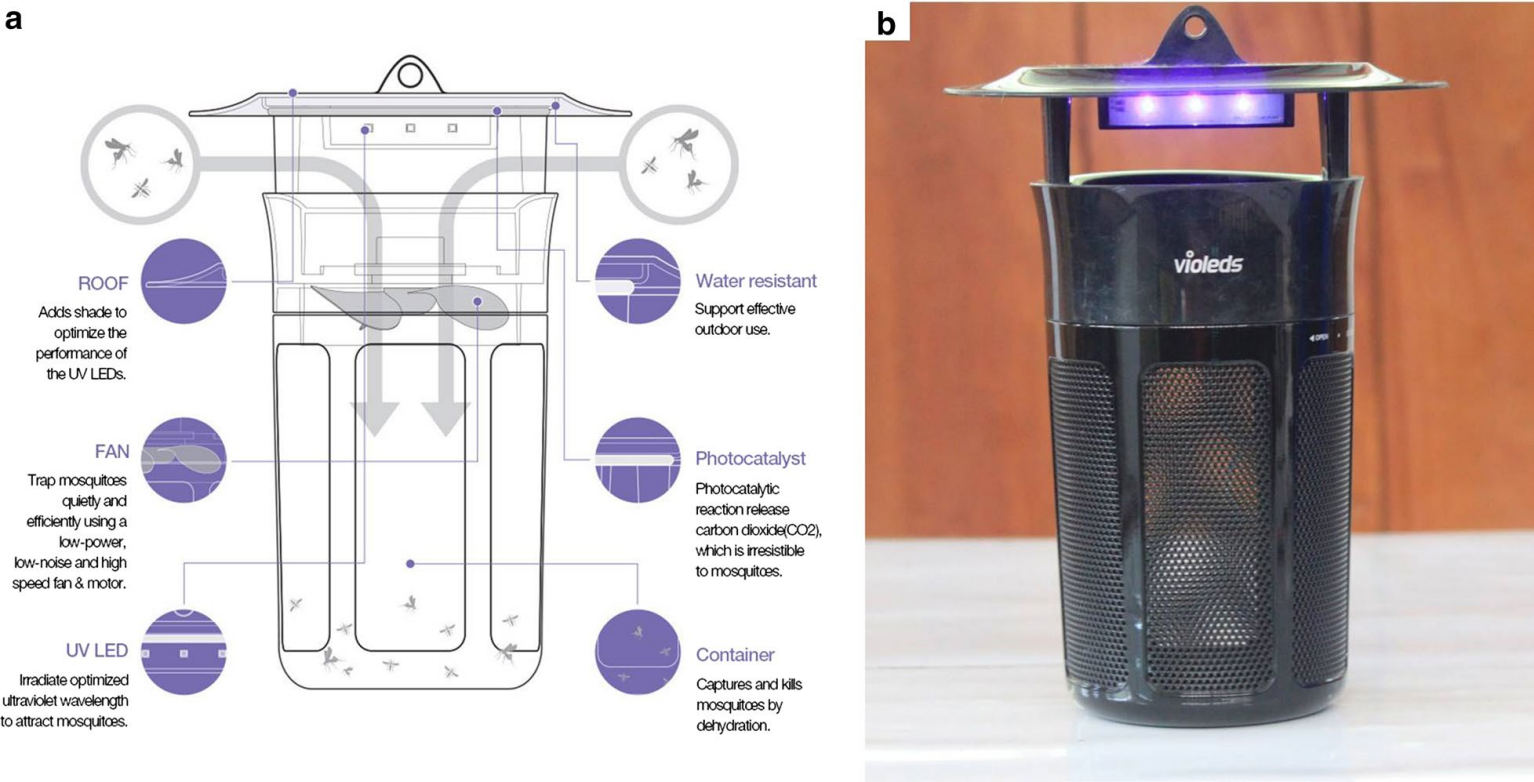
**a** Schematic view of the Mosclean trap. **b** Close-up picture of the Mosclean trap. Schematics courtesy of Seoul Viosys [34]

As shown in Fig. 2a, the trap has five components: (i) roofing plate, which has a suspension tag for hanging the trap and a shade to optimize performance of the UV LEDs; (ii) a low-power, low-noise and high efficiency fan for mosquito suction; (iii) a UV LED plate, emitting ultraviolet light at a wavelength of 365 nm to attract mosquitoes; (iv) a container for capturing trapped mosquitoes; and (v) a photocatalyst, which according to the manufacturer releases CO_2_ gas to complement mosquito attraction. We did not test the CO_2_ production by these traps during our experiments, but instead evaluated the traps just as obtained from the manufacturer.

The trapping efficacy of Mosclean trap was compared against those of other existing traps commonly used for sampling indoor and outdoor *Anopheles* and culicine mosquitoes. The first trap was the CDC-light trap, widely used for trapping indoor mosquitoes [19, 20]. The CDC-light trap uses an incandescent light bulb as the main attractant, and runs on a motorized fan powered by a battery [19, 20]. The second was the BG-Sentinel trap (Biogents GmbH, Regensburg, Germany) [15, 38]. The trap consists of a dark blue collapsible bucket, a white perforated lid, an intake funnel, a catch bag and a ventilator powered by a battery [23]. It is 36 cm in diameter and 40 cm in height. The last comparator trap was the Suna trap (Biogents GmbH), which is 52 cm in diameter and 39 cm in height, and also operates on battery power. The Suna trap was described and optimized for the first time in western Kenya in 2014 [22] and demonstrated high efficacy against *An. funestus* mosquitoes in Rusinga Island [39]. Recently it was used in Malawi to sample mosquitoes indoors and outdoors [40].

Both the BG-Sentinel and Suna traps are commonly used for sampling mosquitoes outdoors, and are often baited with proprietary BG lure, CO_2_ gas or other lures [27, 28, 41]. In this study, CO_2_ was obtained, whenever needed, from yeast-molasses fermentation, and used in the different comparator traps as detailed below. The yeast-molasses mixture was prepared 30 minutes prior to starting the experiments by mixing 40 g of baker’s yeast and 500 ml of molasses dissolved in 2 L of water, and the effluent gases channeled *via* plastic tubing to the traps [42]. As a standard reference, HLCs, performed by adult male volunteers, was also used in semi-field tests, but not in the field tests as the wild mosquitoes might be infectious.

### Study procedures

#### Semi-field tests to compare trapping efficacies of the Mosclean trap, CDC-light trap, human landing catches (HLC), BG-Sentinel trap and Suna trap

The experiments were conducted inside the semi-field compartments each night from 18:00 to 06:00 h. Each night, 400 nulliparous female laboratory-reared *An. arabiensis* aged 3–6 days and not previously blood-fed were released inside the screen house chamber [43]. The test mosquitoes were starved for 6 h prior to each test and were released inside the chambers 30 min before starting the tests to acclimatize in the environment. The huts were cleaned each morning (using a Prokopack aspirator) and traps monitored for optimal functionality.

(i) *Tests to compare the Mosclean trap and CDC-light trap using a human host under a bednet as bait*

**Fig. 3:**
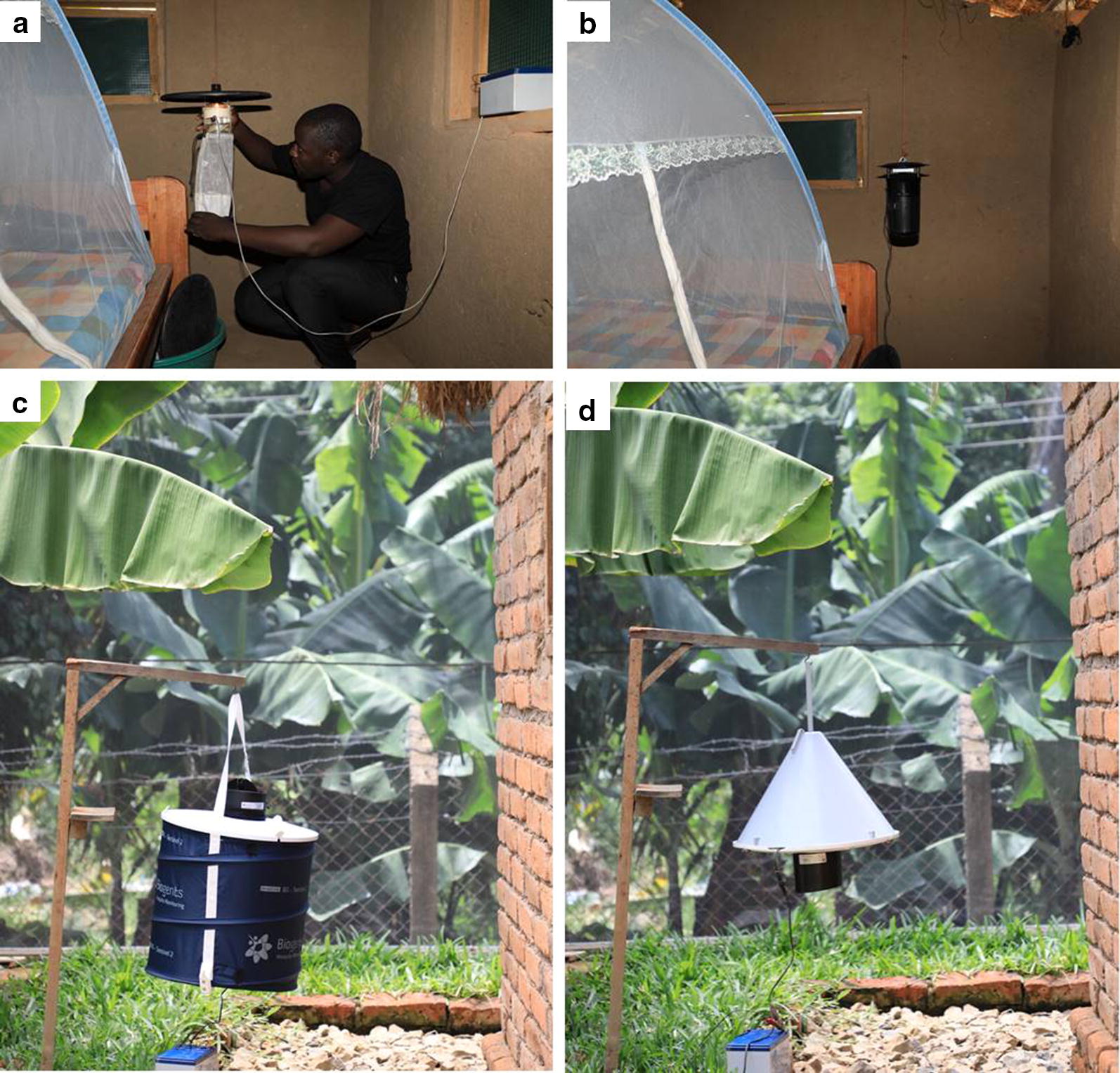
Images of trap set up during semi-field experiment, **a** CDC-Light trap set up indoors beside a bed with a bednet. **b** Mosclean trap indoors beside a bed with a bednet. **c** BG-Sentinel trap set up outdoors. **d** Suna Trap set up outdoors

First, we compared the Mosclean trap against the CDC-light trap for indoor trapping in tests with the two traps inside the same chamber on the same nights, such that the traps were competing for the same mosquitoes. One Mosclean trap was suspended inside one of the experimental huts, beside an untreated bednet occupied by a sleeping adult male volunteer (Fig. 3b). Similarly, one CDC-light trap was suspended inside the other hut, also beside a volunteer-occupied bednet (Fig. 3a). The traps were both 150 cm above ground and close to the volunteers’ feet [20]. The CDC-light trap and Mosclean trap were rotated between the huts nightly to avoid positional bias. The volunteers, however, retained their positions such that their individual differential attractiveness was combined to the respective hut characteristics to constitute a single source of experimental variation. Each night, 400 female *An. arabiensis* mosquitoes were released from a central location inside the semi-field chamber, equidistant from the two huts. Total numbers of mosquitoes caught in each trap overnight was recorded and the traps cleaned. The test was repeated over 12 nights, each trap being in each hut six times.

(ii) *Tests to compare the Mosclean trap and HLCs using human host under a bednet as bait*

The second test compared the Mosclean trap and HLCs, also in a competitive manner. In one hut, a Mosclean trap was set beside a volunteer-occupied net as above (Fig. 3b), while in the second hut, HLC was conducted on the same night by an adult male volunteer. The volunteers sat indoors, folding their trousers to catch mosquitoes landing on their legs using mouth aspirators. This way, the two trapping methods competed for the same mosquitoes. The Mosclean trap and HLC were rotated between the huts nightly, but the volunteers stayed in the same huts. Each night, 400 female *An. arabiensis* mosquitoes were released from a central location inside the semi-field chamber, equidistant from the huts. Mosquito catches by either HLC or Mosclean trap were recorded and compared. The test was repeated over 12 nights, each method being in each hut six times.

(iii) *Non-competitive tests of the Mosclean trap and HLCs, using human host under a bednet as bait*

In the third test, the Mosclean trap was independently tested against HLC on separate nights to avoid direct competition between the traps for the same mosquitoes. The trap was set inside the first hut (randomly selected) then moved to the second hut on the second night. On the third and fourth nights, HLC was conducted inside either the first or second hut in a random order. This sequence was randomized so that the tests rounds began with either HLC or the Mosclean trap, and was also replicated six times. Each night, 400 female *An. arabiensis* mosquitoes were released from a central location inside the semi-field chamber. Numbers of mosquitoes caught each night by each trap type in the absence of a competing alternative were recorded compared.

(iv) *Tests to directly compare the Mosclean trap against commonly used outdoor traps, i.e. Suna and BG-Sentinel traps*

Unlike the first three tests, mosquito trapping in this experiment was conducted outdoors but within the semi-field chambers over four-night cycles using a randomized 4 × 4 Latin square design. Two different variants of the Mosclean trap were used, the first without any additional bait, and the second baited with additional CO_2_ from yeast-molasses fermentation. The Suna trap (Fig. 3d) and BG-Sentinel (Fig. 3c) were also baited with CO_2_ from yeast-molasses fermentation. The four trap types, standard Mosclean trap, CO_2_-baited Mosclean trap, CO_2_-baited Suna trap and CO_2_-baited BG-Sentinel trap were rotated nightly in a random fashion across the four outdoor locations inside the semi-field chamber over four-night cycles. A total of 600 female *An. arabiensis* were released each night at a central location equidistant from each trap position and the catches recorded each morning. The experiment was replicated seven times over 28 nights, during which each trap type had been to each location seven times.

#### Field tests to compare trapping efficacies of the Mosclean trap, CDC-light trap, BG-Sentinel trap and Suna trap

Four different field experiments were conducted as follows:

(i) *Comparison of efficacies of the Mosclean and CDC-light traps for sampling indoor host-seeking mosquitoes*

Four houses were selected randomly in each of the study villages and recruited after obtaining consent from the household heads. In each hut, either a Mosclean trap or CDC-light trap was set beside a bed inside a room where one adult volunteer slept under bednet. Since coverage of insecticide-treated nets was > 90% in these villages, we conducted our study with these rather than untreated nets as used in the semi-field tests. Based on the trap assignment schedule, huts 1, 2, 3 and 4 each received either of the two trap types. No additional lures were added to the traps. The traps (1st Mosclean trap, 2nd Mosclean trap, 1st CDC-light trap and 2nd CDC-light trap) were rotated each night between each of the houses over 12 nights, following a 4 × 4 Latin square design, replicated four times. The tests lasted from 18:00 to 06:00 h each night. Both Mosclean and CDC-light traps were suspended 150 cm above ground, towards the feet of the sleepers. Mosquitoes caught by each trap each night were sorted by taxa, sex and physiological status, then counted and recorded.

(ii) *Comparison of the performance of the Mosclean trap when used indoors and outdoors*

In studies measuring malaria vector biting risk, it is common to assess proportions of biting that occur indoors *versus* proportions that occur outdoors, so as to determine where the biting risk is greater and also whether interventions such as ITNs and IRS are addressing the full spectrum of exposure. Unfortunately, despite recent innovations such as the electric grid traps [17] and human-baited double net traps [18], there are no appropriate trapping systems other than HLCs that consistently and accurately assess these proportions. The Mosclean trap was therefore assessed to determine if it would be suitable for collections both indoors and outdoors. Four households were selected randomly in each village and recruited following consent from the head of household. Mosclean traps were then set either indoors or outdoors at each of the houses. When used indoors, the trap was set beside an occupied bednet. However, when outdoors, the trap was placed approximately 5 m from the house, but next to an adult volunteer sitting under a bednet. The Mosclean trap outdoors was set 5 m from the house, aiming to catch mosquitoes flying close to the human dwellings. The traps were 150 cm above ground, whether indoors or outdoors. Each morning the mosquitoes caught indoors and outdoors were sorted by taxa, sex and physiological status, and the numbers recorded and compared.

(iii) *Tests to compare field efficacies of the Mosclean, BG-Sentinel* and *Suna traps outdoors*

These experiments was conducted outdoors using a 4 × 4 Latin square design in which trap locations were randomly assigned at the start of each round of four sampling nights. Two variants of Mosclean trap were used, one supplemented with CO_2_ gas from yeast-molasses fermentation and another without additional CO_2_. Four locations were identified in each of the two villages, approximately 100 m apart. The four trap types, standard Mosclean trap with no additional bait, CO_2_-baited Mosclean trap, CO_2_-baited Suna trap and CO_2_-baited BG-Sentinel trap, were rotated nightly between the four outdoor locations over four-night cycles. Each morning, mosquitoes caught by each trap were sorted by taxa, sex and physiological status, and the numbers recorded and compared. The tests were performed in the dry season of June 2017 to February 2018.

(iv) *Assessing proportions of parous and proportions of inseminated female Anopheles mosquitoes in Mosclean trap and CDC-light trap catches*

Mosquitoes were sampled in four different houses in one village for 20 nights. All female *An. arabiensis* mosquitoes collected were dissected. Similar trapping was done by CDC-light traps and the mosquitoes dissected under stereo light microscopes so that the parity and insemination rates could be compared.

The dissected ovaries or spermatheca were observed at 10× magnification under a compound microscope. Parity status was confirmed by presence of stretched ovariole tracheoles (parous females) or coiled tracheolar skeins (nulliparous females), as detailed [44]. Insemination was confirmed by observing whether the spermatheca were filled or unfilled [45].

### Data analysis

Data were analyzed using open source statistical software, R v.3.5.0 [46]. The efficacy of the Mosclean trap was compared to those of the other traps by fitting generalized linear mixed modes (GLMM) using the package *lme4* [47]. The number of mosquitoes of different species was modelled following negative binomial distributions, with trap type as the main effect. Experimental days and hut ID were included as a random term to account for unexplained variations within days and huts. Graphs were created in the package *ggplot2* [48]. Logistic regression was used to assess the parity and insemination rates between the CDC-light trap and Mosclean trap. A likelihood ratio test was used to check the effect of random effect.

## Results

### Results of semi-field tests to compare trapping efficacies of the CDC-light trap, human landing catches (HLC), BG-Sentinel and Suna trap

#### Tests to compare the Mosclean trap against the CDC-light trap and HLC

Results for these tests, including nightly mosquito counts and statistical parameter estimates are summarized in Table 1. Additional information is provided in Additional file 1: Figure S1. When placed simultaneously inside the semi-field chambers on the same nights, the Mosclean trap caught twice as many *An. arabiensis* mosquitoes as the CDC-light trap [relative rates (RR) and 95% CI: 2.1 (0.97–4.56)], although the difference was not statistically significant (*P* = 0.059). There was also no statistically significant difference between mean mosquito counts in tests against HLC (RR: 0.74, 95% CI: 0.51–1.07, *P* = 0.113).

**Table 1:** Median number of female *Anopheles arabiensis* mosquitoes recaptured per night by Mosclean trap and other candidate traps inside the semi-field chambers in tests to compare trapping efficacies of the Mosclean trap, CDC-light trap, human landing catches (HLC), BG-Sentinel trap and Suna trap. Table also shows interquartile ranges (IQR) and relative rates (RR)

Experiment	Traps tested	Total no. of mosquitoes recaptured	Median nightly catch (IQR)	RR (95% CI)	*P*-value
Competitive comparison of Mosclean trap and CDC-light trap	CDC-light trap indoors	524	26.5 (20.5–72.75)	Ref	Ref
	Mosclean trap indoors	1102	100.5 (87.75–118.25)	2.1 (0.97–4.56)	0.059
Competitive comparison of Mosclean trap and HLC	HLC indoors	1124	88.5 (82–103)	Ref	Ref
	Mosclean trap indoors	834	79.5 (54.75–96.25)	0.74 (0.51–1.07)	0.113
Non-competitive comparison of Mosclean trap and HLC	HLC indoors	626	102 (98.25–115.50)	Ref	Ref
	Mosclean trap indoors	817	97 (76.75–175.75)	1.1 (0.67–1.82)	0.708
Direct comparison of Mosclean trap against Suna trap and BG-Sentinel trap	CO_2_-baited BG-Sentinel trap outdoors	777	18 (5–27.25)	Ref	Ref
	CO_2_-baited Mosclean trap outdoors	963	32.5 (27.75–42)	1.48 (0.96–2.32)	0.086
	CO_2_-baited Suna trap outdoors	1149	30 (19.50–48)	1.67 (1.08–2.66)	0.021
	Unbaited Mosclean trap outdoors	325	9 (4.0–13.25)	0.46 (0.29–0.72)	< 0.001

#### Non-competitive tests of the Mosclean trap and HLC

In tests when the Mosclean trap or HLC were used singly inside the chambers on different nights without any competition, the traps caught similar numbers of *An. arabiensis* per night (RR: 1.1, 95% CI: 0.67–1.82, *P* = 0.708).

#### Tests to directly compare the Mosclean trap to Suna and BG-Sentinel traps

In the comparative evaluation of the Mosclean trap against CO_2_-baited BG-Sentinel trap and CO_2_-baited Suna trap in semi-field conditions, 3214 mosquitoes were recaptured (19.1% of total released over all experimental nights). The proportion of mosquitoes recaptured by the CO_2_-baited Suna trap was 35.7% (1149); CO_2_-baited Mosclean trap, 30% (963); CO_2_-baited BG-Sentinel trap, 24.2% (777); and un-baited Mosclean trap, 10.1% (325). The percentages for each trap were calculated from the total numbers recaptured. The Mosclean trap when baited with additional CO_2_ caught more mosquitoes compared to the BG-Sentinel trap, although the difference was not statistically significant (*P* = 0.086). On the other hand, the CO_2_-baited Suna trap caught approximately 1.5 times as many *An. arabiensis* as the CO_2_-baited BG-Sentinel trap (*P* = 0.021), but a comparable number to the CO_2_-baited baited Mosclean trap (Table 1).

### Results of field tests to compare trapping efficacies of the Mosclean trap, CDC-light trap, BG-Sentinel trap and Suna trap

#### Comparison of efficacies of the Mosclean trap and CDC-light trap for sampling indoor host-seeking mosquitoes

A summary of the field results are provided in Table 2. The Mosclean trap caught approximately the same number of *An. arabiensis* as the CDC-light trap when the traps were set indoors in different huts (RR: 1.18, 95% CI: 0.84–1.50, *P* = 0.242). Recent evidence from these field sites suggest that the *Anopheles gambiae* complex consists entirely of *An. arabiensis* [49, 50], thus we hereafter refer to them as such throughout the manuscript. The Mosclean trap caught significantly less *An. funestus* mosquitoes than the CDC-light trap (RR: 0.62, 95% CI: 0.43–0.89, *P* = 0.009), but was twice as efficacious in catching *Culex* mosquitoes (RR: 2.18, 95% CI: 1.72–2.77, *P* < 0.001).

**Table 2:** Median number of female *Anopheles arabiensis* mosquitoes recaptured per night by Mosclean trap and other candidate traps in rural Tanzanian villages during tests to compare trapping efficacies of the Mosclean trap, CDC-light trap, BG-Sentinel trap and Suna trap. Table also shows interquartile ranges (IQR) and relative rates (RR)

Experiment	Mosquito species	Trapping methods tested	Total no. of mosquitoes collected	Median nightly catch (IQR)	RR (95% CI)	*P*-value
CDC-light trap *vs* Mosclean trap; indoors	*An. arabiensis*	CDC-light trap	5336	17 (5–50)	Ref	Ref
		Mosclean trap	6291	21 (7.75–52.25)	1.18 (0.89–1.56)	0.242
CDC-light trap *vs* Mosclean trap; indoors	*An. funestus*	CDC-light trap	229	0 (0–2)	Ref	Ref
		Mosclean trap	147	0 (0–1)	0.62 (0.43–0.89)	0.009
CDC-light trap *vs* Mosclean trap; indoors	*Culex* spp.	CDC-light trap	9653	40 (15–96.25)	Ref	Ref
		Mosclean trap	22616	75 (33.75–163.75)	2.18 (1.72–2.77)	< 0.001
Mosclean trap indoors *vs* Mosclean trap outdoors	*An. arabiensis*	Mosclean trap outdoor	240	6 (2–11)	Ref	Ref
		Mosclean trap indoor	291	10 (2.75–16.75)	1.35 (0.83–2.22)	0.229
Mosclean trap indoors *vs* Mosclean trap outdoors	*An. funestus*	Mosclean trap outdoor	13	0 (0–0.25)	Ref	Ref
		Mosclean trap indoor	91	1.5 (1–4.25)	6.93 (3.85–12.46)	< 0.001
Mosclean trap indoors *vs* Mosclean trap outdoors	*Culex* spp.	Mosclean trap outdoor	1035	24.5 (16.75–60)	Ref	Ref
		Mosclean trap indoor	2645	69.5 (40–131.25)	2.53 (1.71–3.75)	< 0.001
Outdoor tests to compare four trap types	*An. arabiensis*	BG-sentinel trap	14	0 (0–1)	Ref	Ref
		Mosclean trap	65	1 (0–2.25)	4.36 (1.62–11.72)	0.003
		Mosclean trap + CO_2_	112	1 (0–8)	7.42 (2.85–19.31)	< 0.001
		Suna trap	30	0.5 (0–2)	2.03 (0.75–5.47)	0.163
Outdoor tests to compare four trap types	*Culex* spp.	BG-sentinel trap	1552	76.5 (26.5–88)	Ref	Ref
		Mosclean trap	969	29 (17.25–44.25)	0.59 (0.39–0.91)	0.017
		Mosclean trap + CO_2_	1991	46 (23.75–78.25)	1.08 (1.70–1.67)	0.698
		Suna trap	1711	62.5 (36.75–98.75)	1.08 (0.71–1.63)	0.707

#### Comparison of performance of the Mosclean trap when used indoors and outdoors

When the efficacy of Mosclean trap was assessed for outdoor and indoor use, it caught more *An. arabiensis*, *An. funestus* and *Culex* mosquitoes indoors than outdoors (Table 2 and Additional file 1: Figure S2). However, these differences were statistically significant for only *An. funestus* mosquitoes (RR: 6.93, 95% CI: 3.85–12.46, *P* < 0.001).

#### *Tests to compare field efficacies of the Mosclean trap, BG-Sentinel trap* and *Suna trap outdoors*

In the 4 × 4 Latin square experiments, where all the four trap types were tested outdoors in the villages, the total number of *An. arabiensis* caught was 221. Of these, the CO_2_-baited Mosclean trap caught 50.7% (*n* = 112), the un-baited Mosclean trap caught 29.4% (*n* = 65), the CO_2_-baited Suna trap caught 15.6% (*n* = 30) and the CO_2_-baited BG-Sentinel trap caught 6.3% (*n* = 14) over the 20 test nights. The number of *An. arabiensis* caught by the un-baited Mosclean trap was four times more than the CO_2_-baited BG-Sentinel (*P* = 0.003), and this increased to seven times when CO_2_ was added to the Mosclean trap (*P* < 0.001).

Concurrently, 6223 *Culex* spp. mosquitoes were caught, of which the CO_2-_baited Mosclean trap caught 32% (*n* = 1991), CO_2_-baited Suna trap caught 27.5% (*n* = 1711), CO_2_-baited BG-Sentinel trap caught 24.9% (*n* = 1552) and un-baited Mosclean trap caught 15.6% (*n* = 969). For this genus, there were no significant differences between the traps (Table 2).

### Proportions of parous and inseminated female *An. arabiensis* in Mosclean trap and CDC-light trap catches

The proportion of parous *An. arabiensis* females was slightly higher in Mosclean trap collections than CDC-light trap collections, while the proportions inseminated were similar. Of the 181 *An. arabiensis* caught by Mosclean trap, which were dissected, 56.4% were parous and 87.8% were inseminated (Table 3). Of the 251 *An. arabiensis* caught by CDC-light trap, which were dissected, 45.8% were parous and 90.4% were inseminated.

**Table 3:** Parity and insemination rates of *An. arabiensis* mosquitoes collected by Mosclean trap and CDC-light trap indoors in the field setting. Table also shows odds ratios (OR)

Method	Total no. dissected	Proportion parous % (*n*)	OR (95% CI) (*P*-value)	Proportion inseminated % (*n*)	OR (95% CI) (*P*-value)
CDC-light trap indoors	251	45.8 (115)	Ref	90.4 (227)	Ref
Mosclean trap indoors	181	56.4 (102)	2.06 (1.24–3.41) (*P* = 0.005)	87.8 (159)	1.57 (0.61–4.07) (*P* = 0.353)

*Abbreviation: n*, total number of parous or total number of inseminated mosquitoes

## Discussion

To improve surveillance strategies against vector-borne infections, new trapping devices are required that demonstrate high levels of efficacy, field robustness, affordability and scalability. This study evaluated a new LED trap that emits UV light as the primary mosquito attractant. The trap was compared to existing trapping methods including CDC-light traps with incandescent light bulbs, as is commonly used for trapping mosquitoes inside human dwellings. Other traps tested were the BG-Sentinel trap and Suna trap. The tests were conducted both indoors and outdoors, in both semi-field settings against laboratory-reared mosquitoes and field settings against wild mosquito populations. Although the main target was the malaria vector *An. arabiensis*, the field tests also revealed moderate to high efficiencies against other mosquito species. Overall, the Mosclean trap was at least as efficacious as HLC, and twice as efficacious as the CDC-light trap when used indoors for sampling the malaria vectors in the semi-field, although the difference not being statistically significant. Similarly, in the field settings, the trap caught slightly more *An. arabiensis* than CDC-light trap. For *Culex* mosquitoes, the Mosclean trap also caught twice as many as the CDC-light trap.

Mosquito traps employ odor and visual stimuli to activate and attract mosquitoes from a distance [17, 51–54]. Insects typically visualize objects in the ultraviolet, blue and green wavelengths [55]. The Mosclean trap was developed to exploit host-seeking behavior of mosquitoes in relation to both olfactory and visual stimuli. The trap utilizes titanium dioxide (TiO_2_) which, releases CO_2_ by a photocatalyst reaction during the operation. It also emits optimized high efficiency UV LEDs (wavelength of 365 nm) to attract mosquitoes. An additional advantage is that the lamp can run for more than 10,000 hours and therefore requires less frequent replacements than the incandescent lamps used on the CDC-light trap which typically runs for 1200 hours or less [56].

Previous trials in South Korea had also showed that LED traps caught significantly higher numbers of host-seeking mosquitoes compared to traditional light traps [57]. This may indicate the importance of natural human cues, and also host biomass, which directly influence biting densities [58]. In our outdoor-indoor tests, the traps were set near volunteer-occupied mosquito nets. However, there was likely a greater concentration of host cues indoors due to a higher number of people indoors compared to outdoors, resulting in a higher number of catches indoors. In tests where the Mosclean trap was placed either indoors or outdoors, *An. funestus* mosquitoes appeared highly endophilic, just as previously observed by Ngowo et al. [50]. The trap was more efficacious indoors than outdoors for catching this vector species, which now mediates more than 80% of transmission in the study area [49]. Nearly seven times the number of *An. funestus* and nearly two times the number of *An. arabiensis* were caught indoors compared to outdoors.

In this study, the Mosclean trap was also evaluated against HLCs in the semi-field, but not in the field due to ethical concerns. In the semi-field tests with laboratory-reared *An. arabiensis*, the trap was as effective as HLCs in both competitive and non-competitive settings (Table 1). The HLC method has multiple disadvantages which limit its application for field studies. It is labor-intensive and needs close supervision, is expensive and may expose human volunteers to potentially-infectious bites [16]. To address these issues, innovative methods have been developed as substitutes, but many of these alternatives do not completely address the challenges. For example, mosquitoes trapped by the mosquito electrocuting trap (MET) dry quickly, making it unsuitable for scientific investigations, such as dissection for parity and insemination status, which require fresh samples [17]. In Burkina Faso [53], a human decoy trap (HDT) was recently demonstrated to catch significantly higher numbers of *Anopheles* spp., *Culex* spp. and *Mansonia* spp. than HLC. However, HDTs are not easily scalable as they need human volunteers and a supply of boiled water [53], making them expensive to conduct.

The Mosclean trap was also efficient outdoors for trapping *An. arabiensis* mosquitoes, compared to other trapping devices previously used for the same purpose (Table 2). It caught more malaria vectors and had higher trapping efficiencies, which were further enhanced when CO_2_ was added. The Suna trap has been observed to perform well in catching *An. gambiae* in semi-field settings [22] and *An. funestus* in field settings [39], and is therefore considered an effective field trapping technique. In this study, the field-trapping efficiencies of the Mosclean trap exceeded both the BG-Sentinel trap and Suna trap.

*Anopheles arabiensis* mosquitoes caught by the traps were dissected to assess epidemiological importance. This examination revealed that more than half of host-seeking mosquitoes caught by Mosclean trap indoors were parous. The values are comparable to or slightly higher than those obtained in CDC light traps (Table 3). Furthermore, nearly 90% of the females caught indoors were inseminated. Together, these findings suggest that the physiological status, and approximate age of the collected mosquitoes is likely to be similar between the Mosclean trap and CDC light trap. Past studies have shown similar observations of high parity rates among indoor malaria vectors [59].

The version of Mosclean traps used was less expensive than other commercially available traps. For example, the Suna trap and BG-Sentinel trap are listed by the manufacturer at US$ 168.11 per unit [60], while the CDC-light trap is listed at US$ 106.00 per unit [61]. By comparison, the Mosclean trap is available for US$ 67.05 [62]. Because of their higher energy efficiency, Mosclean traps are also expected to use less battery power than the other traps.

One limitation is that the UV-LED technology may trap other non-target organisms when used in field settings, especially outdoors, potentially making the trap less environmentally friendly. Another limitation is that we did not measure the quantities of CO_2_ gas produced by the TiO_2_ component of the Mosclean trap as claimed by the manufacturer. These issues need to be investigated before the trap can be considered as a replacement in regular surveillance programs.

## Conclusions

The UV LED trap (Mosclean trap) demonstrated substantive efficacies for trapping of *Anopheles* and *Culex* mosquitoes, and was better indoors than outdoors. The trap was either comparable with or better than existing comparator traps, and could have potential for mosquito surveillance. It is easier to use, cost-friendly and less noisy during its operation. The trap can also be used indoors near human-occupied bednets (the human acting as non-exposed bait) or outdoors, in which case it is beneficial to add other baits such as CO_2_ gas. Importantly, the Mosclean trap catches mosquito populations of high epidemiological importance, with high proportions of parous and insemination females. However, the potential of LED traps, including the Mosclean trap, to catch non-targeted organisms should be investigated further, especially when used outdoors. Furthermore, more studies should be undertaken to validate these findings in other areas and to assess the efficacy of the Mosclean trap against a greater diversity of mosquito species.

## Supplementary information

**Additional file 1: Figure S1.** Number of *An. arabiensis* mosquitoes recaptured per night in the semi-field experiments to evaluate efficacy of the Mosclean trap against other traps. **a** Mosclean trap tested competitively against CDC light trap indoors, with both traps in the chamber on the same nights. **b** Mosclean trap tested competitively against HLC when both traps are in the chamber on same nights. **c** Mosclean trap tested against HLC, when the traps are set in the chambers individually in different nights. **Figure S2.** Median number of mosquitoes caught per night indoors and outdoors by the Mosclean trap in rural south-eastern Tanzania. Data collected in four houses in 2 villages, over 12 nights for *An. arabiensis* (**a**) and *Culex* spp. (**b**) mosquitoes.

## Data Availability

Data supporting the conclusions of this article are included within the article and its additional files. The dataset generated during this study is available from the corresponding author upon request.

## Abbreviations

HLC: human landing catch
LEDs: light-emitting diodes
UV LED: ultraviolet light-emitting diodes
MET: mosquito electrocuting trap
HDT: human decoy trap
CO_2_: carbon dioxide
