# Supplementary material for: Evaluation of an ultraviolet LED trap for catching Anopheles and Culex mosquitoes in south-eastern Tanzania
Source: Parasit Vectors. 2019 Aug 27;12:418. doi: 10.1186/s13071-019-3673-7 (PMC6712696; doi:10.1186/s13071-019-3673-7)
Supplement: Supplementary file 1 — Additional file 1: Figure S1. Number of An. arabiensis mosquitoes recaptured per night in the semi-field experiments to evaluate efficacy of the Mosclean trap against other traps. a Mosclean trap tested competitively against CDC light trap indoors, with both traps in the chamber on the same nights. b Mosclean trap tested competitively against HLC when both traps are in the chamber on same nights. c Mosclean trap tested against HLC, when the traps are set in the chambers individually in different nights. Figure S2. Median number of mosquitoes caught per night indoors and outdoors by the Mosclean trap in rural south-eastern Tanzania. Data collected in four houses in 2 villages, over 12 nights for An. arabiensis (a) and Culex spp. (b) mosquitoes. [file 13071_2019_3673_MOESM1_ESM.docx]

**Additional file 1**

**
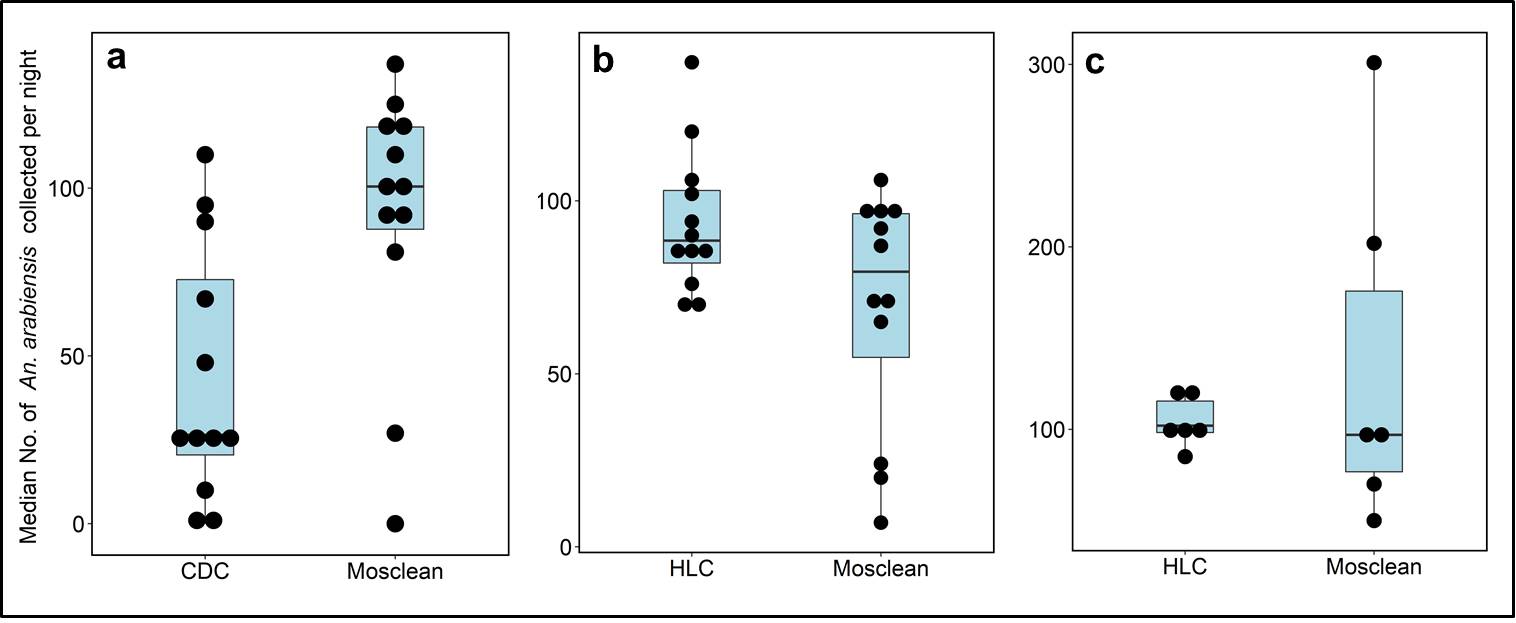
**

**Additional file 1: Figure S1.** Number of *Anopheles arabiensis* mosquitoes recaptured per night in the semi-field experiments to evaluate efficacy of Mosclean trap against other traps: (a) Mosclean trap tested competitively against CDC light trap indoors, with both traps in the chamber on the same nights; (b) Mosclean trap tested competitively against HLC when both traps are in the chamber on same nights; (c) Mosclean trap tested against HLC, when the traps are set in the chambers individually in different nights.


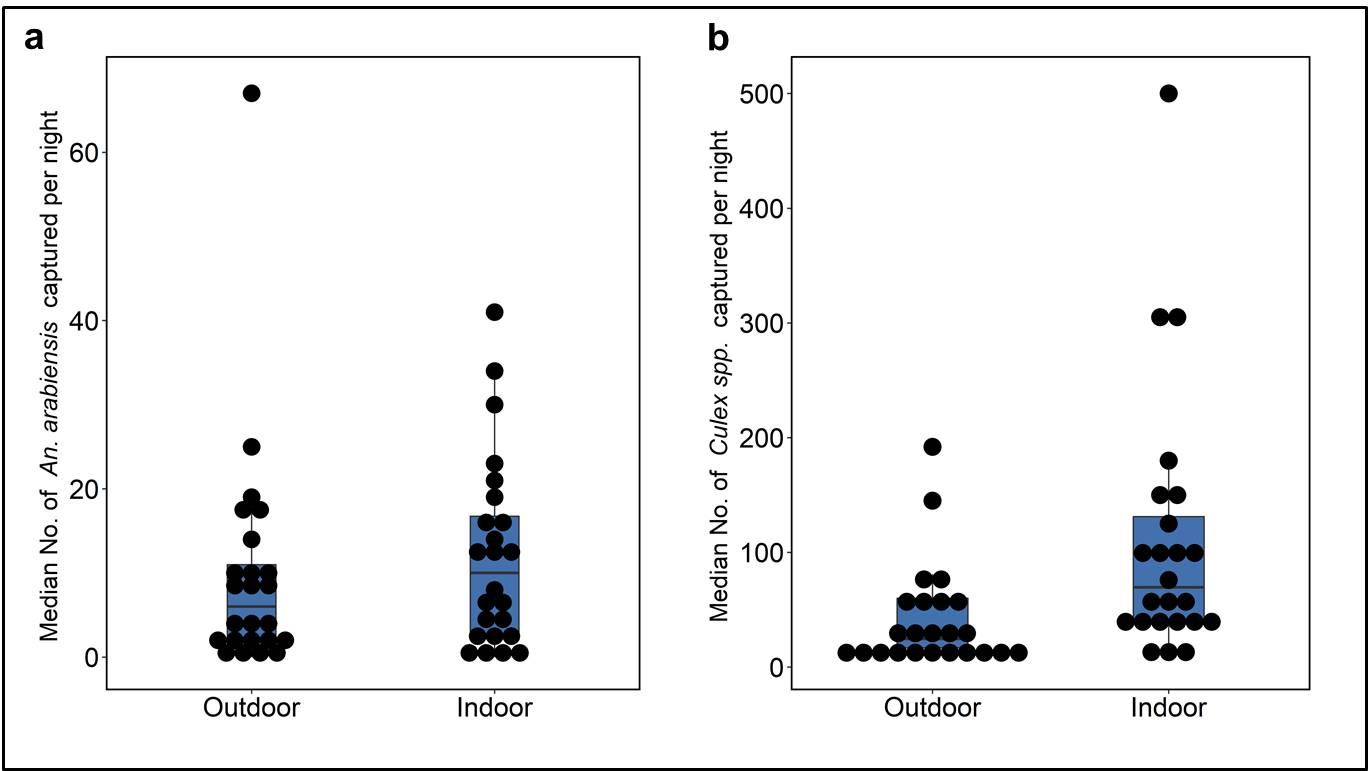


**Additional file 1: Figure S2.** Median number of mosquitoes caught per night indoors and outdoors by Mosclean trap in rural south-eastern Tanzania (Data collected in four houses in 2 villages, over 12 nights for *Anopheles arabiensis* (a) and *Culex* spp. mosquitoes (b)).
